# Acromegaly and Vaccination

**Published:** 1899-08

**Authors:** M. R. Leverson

**Affiliations:** Fort Hamilton, N. Y.


### ACROMEGALY AND VACCINATION.

M. R. Leverson, M. D., Fort Hamilton, N. Y.

If the case of Mary Fogarty, mentioned in the New York *World* of July 21st, be investigated, I predict that it will be found that her "acromegaly" (*i. e.*, excessive growth of bone) began to develop within a few years, probably within two years, after she was last vaccinated.

The disease of acromegaly—or osteomegaly, was unknown until the rite of vaccination became common.

The connection of cause and effect between the two follows clearly from the known laws of biology, or the science of life-growth.

The same laws of biology explain the connection between vaccination and the enormous increase in cancer in recent times. The same laws of biology account for the terrible increase in tuberculosis from the same cause, though the learned men recently in Congress in Berlin were unable to see it; it was too plainly before their eyes. "They could not see the forest for the trees." "Yankee Doodle came to town upon a little pony. He said he could not see the town; there were so many houses."

